# Iteration of Tumor Organoids in Drug Development: Simplification and Integration

**DOI:** 10.3390/ph18101540

**Published:** 2025-10-13

**Authors:** Rui Zhao, Qiushi Feng, Yangyang Xia, Lingzi Liao, Shang Xie

**Affiliations:** Department of Oral and Maxillofacial Surgery, Peking University School and Hospital of Stomatology & National Center for Stomatology & National Clinical Research Center for Oral Diseases & National Engineering Research Center of Oral Biomaterials and Digital Medical Devices, Beijing 100081, China; zr@bjmu.edu.cn (R.Z.); qiushifeng163@163.com (Q.F.); 1910303119@pku.edu.cn (Y.X.); liaolz1104@163.com (L.L.)

**Keywords:** organoid plus and minus, personalized medicine sieve, interdisciplinary convergent technologies, precision medicine, composite in vitro model

## Abstract

The inherent complexity and heterogeneity of tumors pose substantial challenges for the development of effective oncology therapeutics. Organoids, three-dimensional (3D) in vitro models, have become essential tools for predicting therapeutic responses and advancing precision oncology, with established correlations to clinical outcomes in patient-derived models. These systems have transformed preclinical drug screening by bridging the gap between conventional two-dimensional (2D) cultures and in vivo models, preserving tumor histopathology, cellular heterogeneity, and patient-specific molecular profiles. Despite their potential, limitations in tumor organoid biology, including inter-batch variability and microenvironmental simplification, can undermine their reliability and scalability in large-scale drug screening. To overcome these challenges, the integration of advanced technologies such as artificial intelligence (AI), automated biomanufacturing, multi-omics analytics, and vascularization strategies has been explored. This review highlights the “Organoid plus and minus” framework, which combines technological augmentation with culture system refinement to improve screening accuracy, throughput, and physiological relevance. We are convinced that the future of drug development hinges on the convergence of these multidisciplinary technologies with standardized biobanking and co-clinical validation frameworks. This integration will position organoids as a cornerstone for personalized drug discovery and therapeutic optimization, ultimately advancing the development of efficacy in oncology.

## 1. Introduction

Organoids have emerged as a transformative tool in preclinical oncology, offering significant advantages in faithfully recapitulating key features of primary tumors, including molecular, phenotypic, and histopathological characteristics [1,2]. These three-dimensional (3D) models address the limitations of conventional two-dimensional (2D) cell cultures, which fail to capture the complexity of in vivo tumor biology. In contrast, organoids maintain the architectural integrity, in vivo-like microenvironmental cues, and essential cellular heterogeneity of parental tumors, critical for modeling tumor behavior and therapeutic responses [3,4]. Increasing evidence highlights a strong correlation between therapeutic responses in patient-derived organoids (PDOs) and clinical outcomes, positioning them as valuable predictive platforms for personalized oncology [5,6]. Recent advances in generating reproducible, complex human stem cell-derived metabolic organoids have expanded their applications beyond tumor modeling, including drug toxicity testing, response profiling, and high-throughput screening in metabolic diseases. Additionally, these models enable biobanking of patient-specific organoids, supporting precision medicine initiatives through in vitro pharmacogenomic studies [7].

The structural and metabolic similarities between organoids and native tissues make them highly effective preclinical tools for evaluating drug toxicity and safety [8]. Their rapid generation and scalability further enhance their utility in drug repurposing studies [9]. Notably, PDO-based drug sensitivity assays facilitate patient stratification by identifying genetic or epigenetic signatures correlated with therapeutic efficacy, thus refining precision oncology strategies [10]. In comparison to conventional 2D cultures, organoid systems reduce the occurrence of false-positive drug hits and improve the accuracy of cardiac safety predictions during preclinical screenings [11].

On 11 April 2025, the U.S. Food and Drug Administration (FDA) announced a significant policy shift, outlining plans to phase out traditional animal testing in favor of laboratory-cultured organoids and organ-on-a-chip (OoC) systems for drug safety evaluation. Pharmaceutical companies will be permitted to submit non-animal experimental data derived from these advanced platforms as the basis for regulatory approval. Priority will be given to companies adopting innovative testing methodologies.

Despite their substantial potential in drug screening and therapeutic development, traditional organoid models face significant limitations. These include variability in cell composition, lack of standardization, high costs, scalability challenges, and inter-individual heterogeneity, which impede their broader application in both preclinical and clinical settings [12,13]. A key concern is the inadequate replication of the tumor microenvironment (TME) in organoids, particularly those derived from adult stem cells (aSCs), where stromal and immune co-culture systems are still underdeveloped [14]. The absence of standardized protocols across laboratories results in inter-batch variability, compromising experimental consistency and reproducibility, critical factors for reliable drug screening and therapeutic optimization [15,16]. While vascularization has been observed in transplanted organoids, modeling dynamic stromal intercellular communication and inducing functional angiogenesis within organoid systems remain unresolved challenges. These limitations hinder the development of more complex models necessary for immuno-oncology studies, drug metabolism evaluations, and organ-specific pharmacodynamic assessments [17,18]. Clinical translation is further constrained by small sample sizes and insufficient validation across diverse cohorts, primarily due to limited biopsy availability and inconsistent culture conditions [19,20]. Although aSC-derived tumor organoids generally maintain genomic stability [21,22,23,24], exceptions exist: normal intestinal organoids from pooled crypts may develop clonal dominance during extended culture, complicating genome-wide CRISPR screens [25], and prolonged culture can introduce subclonal mutations, particularly in non-coding regions, that subtly alter drug response profiles and efficacy predictions [26,27]. These challenges underscore the need for enhanced quality control and continued methodological refinement.

Recent technological advancements are progressively addressing the limitations of traditional organoid models. The integration of innovative methodologies with organoid culture systems has the potential to enhance drug screening accuracy, diagnostic precision, and treatment personalization. Engineered matrices now provide spatiotemporal control over morphogenic signals, effectively mimicking dynamic tissue microenvironments and improving developmental robustness [28]. Organoids derived from wild-type stem cells, coupled with phenotype-oriented gene editing, offer new avenues for modeling metastasis and drug resistance [29]. Protocols utilizing aSCs enable rapid in vitro drug response testing, facilitating the development of tailored therapies for patients with rare genetic mutations [30]. Furthermore, transplantable organoid systems, which maintain phenotypic stability following engraftment in animal models, show promise in both regenerative medicine and translational oncology [31,32].

Herein, we propose the “Organoid Plus and Minus” framework, an integrated research strategy that unites organoid technology with interdisciplinary innovation. The core principle of this framework lies in the synergistic realization of two complementary approaches: internal optimization of organoid culture systems, enhancing reproducibility and operability through the rational simplification of culture conditions, and external functional enhancement—augmenting microenvironmental complexity by applying engineering and technological advances. By systematically integrating progress from computational science, engineering, immunology, and vascular biology, this framework expands both the functional scope and translational potential of conventional organoid models. In this context, the “Organoid Plus and Minus” paradigm is expected to facilitate the development of next-generation platforms for precision oncology and pharmaceutical research. Such models more faithfully recapitulate human physiological conditions, markedly improve the accuracy and reproducibility of preclinical drug evaluation, and demonstrate stronger potential for clinical translation. This review, therefore, underscores how the convergence of interdisciplinary methodologies can substantially enhance the performance of organoid models in terms of screening efficiency, scalability, and physiological relevance, thereby accelerating their application as clinically actionable tools in drug discovery and precision oncology.

## 2. Minus in Organoid Culture

The groundbreaking discovery of induced pluripotent stem cells (iPSCs) by Professor Shinya Yamanaka in 2006 represented a pivotal advance in stem cell research. This study demonstrated that enforced expression of four transcription factors (Oct4, Sox2, Klf4, and c-Myc) could reprogram mouse fibroblasts into pluripotent stem cells with properties comparable to embryonic stem cells [33]. This achievement provided the conceptual and methodological foundation for regenerative medicine and disease modeling, ultimately earning Yamanaka the 2012 Nobel Prize in Physiology or Medicine. In parallel, the establishment of organoid systems has further expanded translational opportunities; however, traditional culture conditions based on exogenous cytokines present important limitations. Growth factor–enriched media, while designed to approximate the in vivo microenvironment, can compromise phenotypic stability and reduce translational relevance. In this context, a “minus” strategy, defined by minimizing exogenous growth factors or culturing under physiologically restrictive conditions, has emerged as a promising alternative. Notably, this approach may better preserve tissue-specific characteristics and mitigate confounding factors such as tumor heterogeneity, thereby improving the predictive utility of organoid models for preclinical drug development [34].

Recent advances have established low–growth factor culture systems to overcome the limitations of conventional media. For example, studies on colorectal cancer organoids (CRCOs) have demonstrated that activation of the Wnt and EGF signaling pathways, as well as inhibition of BMP signaling, are not essential for the survival of most CRCOs. A medium formulated without R-spondin, Wnt3A, and EGF not only sustained CRCO proliferation but also preserved the intratumoral heterogeneity of the original samples and generated drug response data with improved predictive validity [34]. Conventional Matrigel, with its undefined composition and endogenous growth factor content, restricts precise control of the culture microenvironment and complicates the evaluation of specific signaling inputs during organoid development [35]. To address these challenges, novel defined biomaterials and engineered scaffolds are being actively developed and increasingly integrated with low–growth factor strategies to provide greater control and reproducibility in organoid culture. In parallel, the use of conditioned medium as a culture supplement has been explored to enhance the establishment efficiency and proliferation of colonic organoids [36]. Conditioned medium, enriched with endogenously secreted growth factors and cytokines, can partially substitute for commercial additives, thereby lowering costs and potentially improving culture reproducibility. Notably, this approach provides a practical framework for refining tumor organoid platforms and enhancing their application in preclinical drug screening.

The application of defined and tunable biomaterials, micropatterning techniques, and 3D bioprinting methods provides several advantages, including enabling spatial guidance for organoid growth and morphogenesis, enhancing the efficiency of cell–cell interactions, and reducing dependence on diffusible growth factors. Moreover, these platforms allow precise regulation of both the type and concentration of supplemented factors, thereby facilitating the rational design of minimal media [35,37]. For instance, Cristina Quílez et al. employed 3D printing technology to fabricate spindle-shaped hydrogel devices that mitigated organoid necrosis and supported stable growth under reduced growth factor conditions [38]. The printed microfluidic devices further enabled precise control of morphogen gradients, accurately directing differentiation while minimizing factor consumption, and successfully promoted the generation of skin organoids from pluripotent stem cells. OoC and microfluidic platforms represent an additional promising avenue for reducing growth factor requirements [39]. These systems provide fine-tuned control of the culture microenvironment, including nutrient and growth factor gradients, thereby decreasing reliance on supraphysiological concentrations of exogenous supplements. In this context, such strategies are expected to enhance the translational relevance of organoid models for pharmacological testing, particularly in preclinical drug development.

In summary, the importance of the “minus” strategy in organoid research is well illustrated through advances in both culture medium systems and cultivation scaffolds. This strategy is broadly recognized as a means to enhance the inductive potential of organoids by employing simplified culture conditions for diverse applications. Nevertheless, current evidence indicates that a reduction in growth factors alone is insufficient to address the increasingly complex requirements of translational research. In this context, the integration of emerging technologies signals the onset of a new “Organoid Plus” phase, which extends beyond minimizing endogenous factors to incorporate the enhancement of exogenous technologies, thereby expanding the applicability of organoid platforms in pharmacological development.

## 3. Computational Approaches in Organoids: Drug Screening and Multi-Omics

The iteration of recognition and mimicry algorithms has revolutionized the evaluation and application of organoid models in precision medicine. By integrating 3D imaging technologies, multi-omics data, and predictive computational frameworks, researchers are addressing longstanding challenges in organoid-based drug discovery and disease modeling. This section highlights advanced methodologies for assessing organoid activity, predicting drug responses, and conducting multi-omics analyses, with a focus on how artificial intelligence (AI) -driven innovations improve the reproducibility, scalability, and clinical translatability of organoid systems. These computational approaches are reshaping organoids into dynamic, high-fidelity platforms for personalized oncology and the study of congenital diseases, offering a robust data source for precision therapeutic strategies.

### 3.1. Organoid Activity Assessment Through Image Recognition

Recent advancements in deep learning (DL) algorithms, such as convolutional neural networks, have led to significant progress in 3D volume replication techniques, particularly within the field of organoid research [40]. These technologies, which excel at analyzing complex 3D structures, are well-suited to the inherent architectural properties of organoids, thereby enhancing their utility in drug screening workflows. Notably, Deininger et al. pioneered the integration of magnetic resonance imaging (MRI) with neural network algorithms for non-invasive monitoring and quality assessment of cerebral organoids [41]. This approach allows for precise organoid identification and establishes a reliable foundation for subsequent drug screening. Furthermore, it offers improved efficacy in evaluating 3D morphological features, providing an advantage over conventional methods that require separate observations at distinct developmental stages.

In parallel, Chawan et al. developed the first expert-annotated, high-throughput organoid image dataset, specifically designed for organoid detection and tracking [42]. Their team further innovated a deep neural network architecture capable of performing two critical functions. The system enables frame-by-frame detection of organoids in time-series data and dynamic tracking through cross-frame similarity matching. This dual-function system represents a significant advancement in high-throughput drug screening, allowing for rapid and precise organoid monitoring throughout culture processes, thereby reducing the workload for researchers. Recent technological developments also include OrgaExtractor, a DL-powered image processing tool developed by Park et al. [43]. This platform facilitates non-invasive cell quantification by measuring the total projected area, enabling the comparative analysis of growth patterns across organoid samples. Additionally, it allows for single-organoid morphological characterization, optimizing culture conditions for individual specimens.

The integration of AI-powered imaging tools with organoid systems has the potential to significantly advance the drug screening process. Future innovations, including real-time tracking and quantitative morphological analysis, could facilitate the development of fully autonomous platforms capable of dynamically mapping drug responses across thousands of PDOs.

### 3.2. Prediction of Drug Response Based on Big Data Models

Recent advancements in machine learning (ML) have enabled multi-dimensional approaches to predicting drug responses. Kong et al. developed a network-based ML framework to identify biomarkers from drug-genome datasets derived from organoid models, followed by experimental validation on these platforms [44]. By integrating post-treatment biomarker transcriptomic data with clinical survival outcomes from cancer patients, this approach successfully predicted patient-specific drug responses. Notably, the identified biomarkers were validated in both drug-sensitive and drug-resistant genetically engineered cancer cell lines, substantially improving the predictive accuracy of organoid-based models for personalized drug response [44].

Conventional drug screening systems are hindered by their inability to replicate the complexity of tissue microenvironments, leading to suboptimal drug efficacy evaluations. Transcriptome sequencing provides valuable mechanistic insights into drug actions [45,46]. The development of organoid-based drug sensitivity transcriptome databases addresses the limitations of traditional 2D cell line data, offering robust datasets for the creation of ML models to predict drug efficacy [47]. The integration of AI with organoid technology has transformed drug response prediction by combining ML-driven biomarker identification, such as network-based frameworks validated in organoid and cell line models, high-fidelity data from organoid and OoC systems, and AI-powered analysis of multi-omics big data, including imaging, transcriptomics, metabolomics, and proteomics.

### 3.3. AI Enhances Multi-Omics Analysis in Organoid Research

Multi-omics data analysis, encompassing genomics, metabolomics, and single-cell omics, is critical for evaluating organoid functionality, providing a comprehensive characterization of their molecular landscapes [42,48]. Compared with directly analyzing biopsy samples, organoid-based multi-omics offers distinct advantages. Clinical biopsy specimens are often limited in availability and quantity, which restricts their utility for extensive clinical research. In contrast, organoids can be reliably expanded in culture while preserving key genetic features over several passages, thereby generating sufficient material for sequencing across multiple omics platforms [49]. Moreover, biopsy samples are restricted to observational analyses and cannot capture the effects of experimental interventions, while organoids enable the investigation of dynamic responses to pharmacological perturbations, including drug treatments [50]. Importantly, biopsy-derived data typically reflect a single temporal state, whereas organoids can recapitulate the entire disease trajectory from a developmental perspective, thereby facilitating the temporal reconstruction of pathological processes [51]. Despite these advantages, the integration of heterogeneous multi-omics datasets remains a major challenge, as they encompass unstructured, semi-structured, and high-dimensional data types. The resulting large-scale datasets frequently exceed the analytical capacity of conventional computational approaches [48].

AI algorithms present a transformative approach for the efficient processing of multi-omics data derived from organoids. These technologies enable the detailed analysis of complex biological processes, including dynamic gene expression patterns, proteomic networks, and metabolic pathways [42,48]. Moreover, AI-driven pharmacogenomic analyses hold significant potential for personalized oncology, where drug response prediction models can optimize treatment regimens by linking anticancer agents to patient-specific tumor profiles, thereby minimizing ineffective therapies and reducing adverse effects [48].

Advances in multi-omics approaches, including genomics, transcriptomics, proteomics, epigenomics, and metabolomics, when applied to heart organoids, combined with recent developments in AI, enable the integration of diverse datasets for a comprehensive understanding of cardiac development at multiple levels. AI-driven tools, such as ML and DL, enhance the processing of sequencing data from heart organoids by merging genomic, epigenomic, transcriptomic, and clinical datasets. This integration aids in identifying genetic variants and molecular signatures associated with developmental mechanisms and disease susceptibility. Furthermore, combining single-cell sequencing of organoid-derived cells with AI-powered analysis facilitates the decoding of intricate regulatory networks involved in cardiac development, thereby improving the interpretation of multi-omics data to elucidate the pathogenic mechanisms underlying congenital heart defects [52]. Zhang et al. established OrgXenomics, a proteomic database that systematically documents organoids and patient-derived xenograft (PDX) models. This resource provides comprehensive protein expression profiles, functional annotations, and raw datasets across diverse disease contexts, positioning it as a valuable repository for organoid-based proteomic studies [53]. The platform has the potential to support more accurate patient stratification and tailored treatment selection, ultimately enhancing clinical outcomes for precision medicine.

However, most studies remain limited to single-omics approaches with minimal integration of AI. To fully harness AI’s potential, it is essential to advance cross-modal data fusion, dynamic pathway modeling, and biomarker validation. The integration of AI and organoid technology represents a paradigm shift in preclinical research, providing powerful tools for analyzing complex biological systems. For example, image recognition facilitates real-time organoid monitoring, while ML-based models connect multi-omics data to clinical outcomes. AI also addresses data heterogeneity, unveiling disease and therapeutic mechanisms. Nevertheless, challenges remain, including incomplete cross-modal data harmonization and an overreliance on single-omics datasets. Future research should prioritize the development of unified AI frameworks for multi-omics modeling and validation, as this approach will accelerate the translation of organoid models into oncology and regenerative medicine applications. Computational technology is one of the core pillars of Organoid Plus.

## 4. Automation and High Throughput Enable Organoids to Be Used in Disease Treatment

The advancement of organoid technology as physiologically relevant preclinical models hinges on overcoming persistent challenges related to standardization and scalable automation [54]. Current limitations, including batch-to-batch heterogeneity and inefficiencies in manual workflows, underscore the urgent need for integrated engineering solutions. This section highlights transformative strategies aimed at addressing these barriers. By synergizing engineering innovations with biological precision, these approaches seek to establish organoids as robust, high-fidelity platforms for drug discovery and personalized medicine, while simultaneously addressing critical gaps in functional maturation and reproducibility [54,55].

### 4.1. Development of Integrated Systems for Organoid Fabrication

Organoids consistently display significant heterogeneity in morphological characteristics, cellular spatial organization, and population dynamics, even among specimens derived from identical production batches. Overcoming these challenges through enhanced engineering of cellular and morphological complexity, as well as optimization of maturation processes, is essential for developing physiologically relevant, tissue-mimetic systems. Recent advancements in automated culture platforms and high-throughput analytical techniques have substantially improved both the standardization of organoid production and the systematic quantification of developmental parameters.

Ma et al. developed a microfluidics-integrated 3D printing system that templates cell suspension precursors into uniformly spaced microdroplets for organoid production [56]. This system facilitates the high-throughput generation of morphologically consistent organoids, while ensuring precise spatial distribution within microfluidic channels. The resulting organoids can be encapsulated within customizable microfluidic reactors, enabling controlled fluid perfusion for dynamic drug and chemical delivery. To further enhance drug screening, OoC devices, which combine microfluidic technology to simulate dynamic microenvironments, now enable parallelized, high-throughput screening of organoids. For instance, a droplet-based microfluidic chip can generate organoids and evaluate drug responses within 14 days [57].

Given the inherent spatial and temporal heterogeneity of intracellular parameters, such as DNA/RNA sequences [58], protein and exosome expression profiles [59], and organoid structural complexity [60], the integration of AI with microfluidics-enhanced organoid platforms offers substantial potential. This synergistic approach could advance OoC technologies [61] by incorporating real-time multimodal sensing modules to monitor organoid dynamics. Such innovations would enable precise investigations into pharmacokinetic and pharmacodynamic processes, including drug transport, metabolism, toxicity, and therapeutic efficacy.

To address the challenges of standardization and scalability, Königer et al. introduced the Robotic Enabled Biological Automation (ReBiA) system, which utilizes dual-arm robotics to standardize laboratory workflows within a controlled automation environment [62]. By converting manual protocols into automated processes, ReBiA minimizes process-specific developmental requirements, thereby enhancing the cost-effectiveness and reproducibility of in vitro tissue models. Comparative analyses have demonstrated that ReBiA-generated models exhibit morphology, protein expression patterns, and cell viability comparable to manually prepared and native tissues.

Recent advancements have improved organoid standardization and reduced inherent heterogeneity. Future progress will depend on the integration of several key technologies: automated platforms with real-time sensing for dynamic monitoring, AI-driven analytics to accelerate optimization, and customizable microfluidics to enhance physiological fidelity.

### 4.2. Precision-Manufactured Models and Automated Detection Platforms

PDOs have emerged as transformative preclinical models, accurately recapitulating the histological and molecular features of native tumors, thereby enabling high-fidelity drug response profiling. However, conventional PDO generation is hindered by critical limitations. The loss of endogenous stromal and immune components during culture compromises the TME’s complexity, while manual tissue processing introduces fragment size heterogeneity, thereby impeding reproducible high-throughput screening [63,64]. Droplet microfluidic technologies offer a promising solution by providing precise control over the microenvironment through nanoliter-scale fluid manipulation. This approach enables the automated generation of 3D assembloids with tunable cellular composition and standardized sizing [65], thus establishing more physiologically relevant platforms for precision oncology applications.

Zhang et al. developed a patient-specific lung cancer assembloid (LCA) model using droplet microfluidic technology coupled with a microinjection strategy [55]. This platform facilitates the high-precision manipulation of clinical microsamples and the high-throughput generation of LCAs, achieving excellent intra-batch consistency in size, cellular composition, and drug response profiles. The LCA model addresses key limitations of conventional approaches, including the progressive loss of endogenous stromal and immune cells during culture [66,67], as well as the heterogeneity in fragment sizes caused by manual tissue mincing [55,68,69], which impair reproducible high-throughput drug screening.

Despite progress, automating complex manual workflows remains a significant challenge. Robotic systems continue to face limitations in replicating human-level dexterity, particularly in tasks requiring advanced hand-eye coordination. To address this, Boussaad et al. developed a fully automated, multifunctional platform capable of supporting various cell culture modalities, such as 2D nasopharyngeal carcinoma cultures and 3D mesencephalic organoids, while performing parallel high-throughput screenings [70]. This system’s versatility meets the demands of translational medicine by enabling patient-specific precision drug discovery, target-agnostic compound screening, and multi-parametric readout analyses.

The integration of patient-specific assembloid models with automated microfluidic platforms represents a paradigm shift in translational oncology, addressing long-standing challenges related to throughput and reproducibility [64,65]. Adaptive robotics now enables standardized molecular screening across various culture modalities [71], thereby accelerating the transition from clinical sampling to therapeutic validation. Recent advancements in automated organoid fabrication and vascularization have enhanced the physiological relevance, standardization, and functional consistency of these systems. However, challenges persist, including the replication of human dexterity in robotics [70], and the maintenance of stromal-immune interactions in long-term cultures [66]. Future research should prioritize the development of adaptive automation for dynamic microenvironment modulation [61] and vascular perfusion systems that can emulate systemic drug responses [54]. Such innovations will improve pharmacokinetic predictive validity [61] and facilitate the translation of organoid models into precision oncology and regenerative medicine [55,62].

## 5. Immune Co-Culture Systems for Organoids

Organoid technology, which replicates organ development and pathological processes through 3D culture, has significantly advanced tumor immunology research. Conventional 2D cell models are limited in their ability to replicate the intricate cellular interactions within the TME. In contrast, organoids, which integrate immune cells, stromal cells, and tumor cells, generate a more physiologically relevant model that closely approximates in vivo conditions. This system offers valuable insights for drug screening and therapeutic development [64,72,73].

### 5.1. Classical Organoid-Immune Co-Culture Technologies

Classical co-culture methods, widely employed to investigate tumor-immune interactions, include direct co-culture, Transwell-based indirect systems, and air-liquid interface (ALI) culture. Direct co-culture facilitates direct contact between organoids and immune cells, e.g., CD8^+^ T cells, NK cells, making it particularly suitable for studying cytotoxicity. In these protocols, dissociated organoids are co-suspended with immune cells in matrices, e.g., basement membrane extract (BME), or low-adhesion systems, as demonstrated in mucosal melanoma, where CD8^+^ T cell-mediated tumor eradication was observed [74], and cholangiocarcinoma, where T cell-driven elimination occurs through contact and soluble factors [75].

Transwell systems, which physically separate organoids and immune cells, are employed to study paracrine signaling, revealing mechanisms such as CCL5-mediated gemcitabine resistance in pancreatic cancer [76] and contact-independent tumor cell elimination by Jurkat cells [77]. However, these systems lack spatial fidelity [78]. ALI culture, adapted from epithelial models, utilizes Transwell membranes to maintain native immune components, such as tumor-infiltrating lymphocytes (TILs), in tumor organoids derived from colorectal, renal, and lung cancers [79] supporting immunotherapy evaluation, e.g., PD-1/PD-L1 blockade. Nonetheless, ALI culture faces challenges, including progressive immune cell depletion in long-term cultures [79].

### 5.2. Advanced Organoid-Immune Co-Culture Technologies

Advanced approaches aim to recapitulate the complex crosstalk within the TME, extending beyond basic co-culture models by integrating multiple cell types or optimizing the preservation of native components. Multicomponent co-cultures combine organoids with stromal and immune cells. For example, a gastric cancer tri-culture model, organoids/cancer-associated fibroblasts (CAFs)/natural killer (NK) cells, demonstrated that CAFs induce NK cell ferroptosis through iron transfer [80]. Short-term co-cultures, which preserve endogenous immune components within ovarian tissues, facilitated the in vitro evaluation of anti-PD-1/PD-L1 bispecific antibodies [81].

Systems that maintain autologous components include direct co-cultures using alginate cold gel to study breast cancer organoid-tumor-associated macrophage (TAM) interactions, which showed accelerated proliferation, enhanced stemness, and upregulation of pro-tumorigenic pathways [82]. Furthermore, autologous tumor organoid-peripheral blood lymphocyte (PBL) co-cultures enriched tumor-reactive T cells from mismatch repair-deficient colorectal and non-small cell lung cancer patients, amplifying previously undetectable populations [83].

With advances in tumor immunology, the understanding of the complex interactions between tumors and the immune system has substantially deepened. Tumor organoid–immune cell co-culture models provide a robust platform for systematically elucidating how these interactions shape immune responses, thereby establishing a theoretical basis for the rational design of novel therapeutic strategies for cancer patients [72]. Notably, the integration of co-culture methodologies with multi-omics analyses and artificial AI tools, such as the OrganoIDNet platform developed by Nathalia et al., enables real-time monitoring of immunotherapy responses within organoid–immune cell systems. This combined approach yields dynamic insights into treatment-related behaviors of peripheral blood mononuclear cells (PBMCs) under co-culture conditions and demonstrates potential for evaluating the efficacy of immunotherapeutic agents in patient-derived pancreatic ductal adenocarcinoma organoids [84]. In this context, such models facilitate the optimization of personalized treatment strategies and provide an efficient methodological framework for advancing tumor immunology research and the development of innovative immunotherapies.

### 5.3. Applications in Clinical Translation

Organoid-immune co-cultures provide a rapid platform for predicting the efficacy of immune checkpoint inhibitors (ICIs). Zhou et al. demonstrated patient-specific cytotoxicity in cholangiocarcinoma organoids co-cultured with autologous PBMCs and T cells, showing reduced viability and apoptotic marker release [75], with similar results validated in colorectal and non-small cell lung cancer models [85]. For chimeric antigen receptor T-cell (CAR-T) therapy, hepatocellular carcinoma (HCC) organoid models revealed enhanced cytotoxicity of CD39^+^ CAR-T cells targeting hepatitis B virus (HBV) antigens, which was further potentiated by PD-1/Tim-3/Lag-3 knockdown [86]. Additionally, neuroblastoma organoid-CAR-T systems have proven valuable for therapeutic evaluation [87]. Dijkstra et al.’s autologous tumor organoid-PBL co-culture system enriched tumor-reactive T cells from mismatch repair-deficient cancers, amplifying previously undetectable populations to investigate neoantigen immunogenicity and enhance immunotherapy, facilitating longitudinal patient monitoring [83,85].

**Figure 1:**
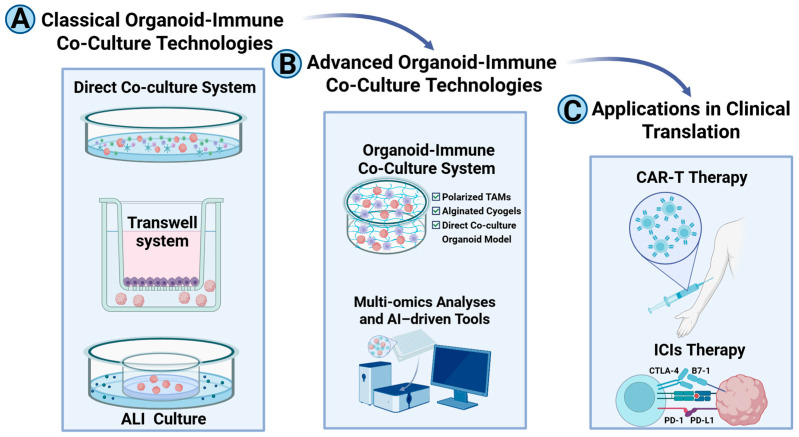
Organoid-immune co-culture technologies and translational applications. (**A**) Classical organoid–immune co-culture technologies. Conventional approaches include direct co-culture, Transwell-based indirect systems, and ALI models. (**B**) Advanced organoid–immune co-culture technologies. Recent innovations incorporate multi-component cultures, preservation of autologous immune components, and hybrid methodologies, collectively enhancing the physiological relevance of co-culture systems. (**C**) Applications in clinical translation. These organoid–immune platforms enable clinically relevant applications, including the prediction of responses to ICIs, assessment of CAR-T cell therapy efficacy, and facilitation of patient-specific immunotherapy screening, thereby strengthening their utility in precision medicine and drug development.

As shown in Figure 1, Organoid-immune co-culture systems, which more accurately recapitulate the complexity of the TME compared to traditional models, utilize techniques such as direct co-culture, Transwell systems, and ALI culture. These systems are critical for investigating both innate and adaptive immune modulation mechanisms and play a pivotal role in predicting the efficacy of therapies, including immune checkpoint inhibitors (ICIs) and CAR-T therapies. Additionally, they facilitate clinical translation, such as individualized treatment monitoring. However, challenges remain, including the limited stability of long-term cultures and incomplete representation of vascular and neural cues. Future efforts should focus on optimizing these systems by integrating real-time monitoring, AI analytics, and other advanced technologies to overcome these limitations and enhance their clinical applicability.

## 6. Vascularizing Organoids

The integration of functional vasculature into organoids represents a significant advancement in preclinical modeling. Traditional organoids face considerable limitations due to the lack of perfusable vascular networks. Organoids exceeding a few hundred microns in diameter, in the absence of adequate vascularization, develop hypoxic cores, leading to central necrosis and impaired cellular function [88,89]. This limitation significantly reduces their physiological relevance for modeling human diseases and drug responses. The vasculature plays a crucial role in oxygen and nutrient exchange, waste removal, and endocrine signaling, key processes that are essential for tissue maturation and homeostasis [90,91]. Vascularized organoids effectively replicate the blood-tissue barrier, providing a platform to study drug extravasation, endothelial cell (EC) interactions, and systemic toxin clearance [92,93].

### 6.1. Stem Cell Co-Differentiation

Organoids derived from adult or pluripotent stem cells generally lack vascular structures. Recent advances in signaling pathway modulation have enabled vascularization through microenvironmental regulation. For instance, Jian Hui Low et al. controlled glomerular-to-tubular ratios by modulating WNT signaling, which dose-dependently upregulated vascular endothelial growth factor A (VEGF-A) to promote the vascularization of human pluripotent stem cell (hPSC)-derived kidney organoids [94]. Similarly, Nazmiye Celik et al. achieved bone organoid vascularization by transfecting adipose-derived stem cells (ADSCs) with miR-210 mimics [95]. Notably, Bilal Cakir et al. demonstrated that human ETS variant 2 (hETV2)-mediated EC reprogramming establishes functional vascular networks in human cortical organoids (hCOs) [96]. These studies collectively highlight stem cell co-differentiation strategies for organ-specific vascularization, establishing co-differentiation as a self-organizing mechanism for generating intrinsic vascular networks across diverse organ types.

### 6.2. Mixed Cell Co-Culture

Vascularization can be enhanced by co-culturing organoid-forming stem cells with vascular progenitors. Due to their well-characterized role in vasculogenesis, human umbilical vein endothelial cells (HUVECs) are commonly employed in such models [97,98]. For example, Yingchao Shi et al. developed long-term viable (>200 days) vascularized brain organoids by co-culturing human embryonic stem cells (hESCs) or human induced pluripotent stem cells (hiPSCs) with HUVECs [99]. Functional enhancement was further achieved through preconditioning ECs [100] or by incorporating fibroblasts [101]. Valeria V. Orlova et al. generated hPSC-derived dendritic cells (DCs) and pericytes that integrated with the host vasculature in zebrafish xenotransplantation models [102]. Furthermore, mesodermal progenitor cells (MPCs) exhibit broad vascularization potential, as demonstrated by Philipp Wörsdorfer et al. in neural and tumor organoid models [103]. Co-culture systems serve as modular platforms for constructing vascularized organoids through synergistic cellular interactions.

### 6.3. Host-Derived Vascularization

This approach capitalizes on the host’s vascular remodeling capacity following transplantation. Abed AlFatah Mansour et al. demonstrated functional neurovascular integration of human brain organoids grafted into murine brains, utilizing two-photon live imaging [104]. Reiner A. Wimmer et al. achieved stable, perfused vascular networks, ranging from arterioles to venules, in transplanted human vascular organoids [105,106]. Notably, Shengtian Zhao et al. observed host-driven vascular maturation in kidney organoids without exogenous VEGF supplementation, occurring within 2–4 weeks post-implantation [107]. Host-engraftment strategies leverage physiological vascular remodeling processes to achieve functional anastomosis between the organoid and host vasculature.

### 6.4. Decellularization-Recellularization

This bioengineering strategy employs acellular vascular scaffolds to guide organoid vascularization. Pedro M. Baptista et al. developed a Triton X-100-based decellularization protocol that preserves the extracellular matrix (ECM) and vasculature in multiple organs, including the liver, kidney, and lung [108]. Recellularization with hepatocytes resulted in the generation of functional, vascularized liver constructs [109]. This methodology provides anatomically accurate templates for engineering organoid vascular networks. By integrating principles of developmental biology with tissue engineering, this biohybrid approach effectively recapitulates native vascular architectures.

### 6.5. Applications of Vascularized Organoids

Vascularized organoid-on-a-chip systems have significantly advanced the modeling of the blood–brain barrier (BBB). Conventional in vitro BBB models, such as Transwell assays, fail to replicate critical dynamic microenvironmental cues, including shear stress-mediated EC interactions. BBB-on-a-chip platforms overcome these limitations by integrating 3D vascularized organoids with microfluidic perfusion, enabling precise evaluation of transcellular transport mechanisms and the regulation of barrier permeability [110,111].

Beyond neurovascular applications, vascularized organoid technologies are bridging the gap between preclinical animal models and human clinical trials. Engineered multi-organ chip systems, including liver [112], kidney [113], pancreas [114,115], heart [115], intestine [116], and bone/bone marrow-on-a-chip [117], facilitate systemic pharmacokinetic and pharmacodynamic studies through interconnected vascular networks [118]. These systems enable the investigation of drug distribution, metabolism, and clearance across multiple organ systems, thereby enhancing the efficiency of drug development pipelines.

**Figure 2:**
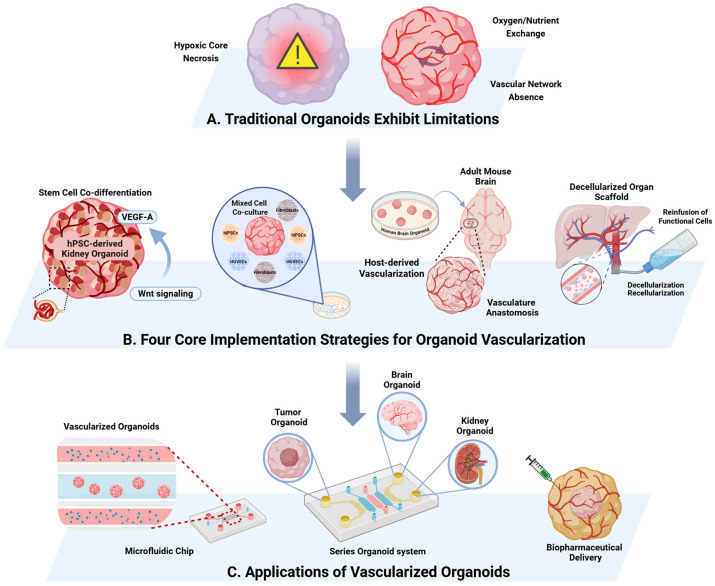
Strategies and applications of vascularized organoids. (**A**) Limitations of traditional organoids. Conventional non-vascularized organoids frequently develop hypoxic necrotic cores due to restricted diffusion of oxygen and nutrients. The incorporation of vascular networks, which more closely replicate in vivo perfusion, mitigates this limitation by facilitating efficient metabolic exchange. (**B**) Core strategies for vascularizing organoids. Four principal approaches have been established to induce vascularization: stem cell co-differentiation, mixed-cell co-culture, host-derived vascularization in vivo, and recellularization of decellularized scaffolds. (**C**) Applications of vascularized organoids. Vascularized organoids serve as advanced platforms for pharmaceutical research. Notably, they enable modeling of the blood–brain barrier under physiologically relevant shear stress, simulation of drug metabolism and pharmacokinetics through interconnected multi-organ chip systems, and the development of tumor models with enhanced translational relevance for studying drug delivery and therapeutic efficacy.

Emerging applications utilize vascularized organoids to study tumor vascular niche-driven metastasis and to develop bioengineered artificial organs. Bioengineered liver organoids, integrated with HUVECs, form vascular networks that accurately recapitulate the TME. Jabri et al. highlighted the utility of vascularized liver organoid models in investigating Sorafenib resistance mechanisms and exploring combination therapies, such as the co-administration of hedgehog signaling inhibitors to enhance Sorafenib sensitivity [119]. This platform also facilitates the evaluation of patient-specific anticancer drug sensitivity [119,120]. These models offer unprecedented resolution for studying cancer-endothelium crosstalk and advancing regenerative medicine through functional vascular integration, which is systematically illustrated in Figure 2.

## 7. Application of Organoid-Related Technologies in Drug Therapy Development

If organoid technologies are confined solely to basic research, they will be insufficient to address the increasing demands of pharmaceutical development. It is therefore essential to integrate “Organoids plus” with applications in drug screening, therapeutic evaluation, and pharmacological as well as toxicological studies. This chapter highlights four key directions previously outlined, with a focus on how “Organoids plus” can overcome critical bottlenecks in drug development, improve the efficiency of compound screening, and enhance the accuracy of efficacy prediction. In this context, these advances hold substantial practical significance and translational value for pharmaceutical research and development.

### 7.1. Application of Computational Approaches in Drug Screening and Efficacy Prediction

As organoid-related technologies increasingly shape the development of drug therapies, the integration of computational approaches into drug screening and efficacy prediction has become particularly critical. These methods enable systematic analysis of complex datasets, facilitate the identification of candidate compounds with optimized pharmacokinetic and pharmacodynamic profiles, and provide predictive insights into therapeutic efficacy prior to preclinical validation.

The Kong team integrated organoid drug–genomic data with clinical survival outcomes to construct a network-based ML model and successfully identified biomarkers associated with chemotherapeutic drug sensitivity. Notably, in rectal cancer, BH3-only proteins function as apoptosis initiators that mediate 5-fluorouracil (5-FU)-induced cell apoptosis. By leveraging this mechanism, patient prognosis can be predicted with greater accuracy, thereby advancing biomarker discovery in oncology [44]. In parallel, advanced image analysis tools have become equally important in this field. For example, OrgaExtractor employs a DL framework based on a multi-scale U-Net architecture to achieve precise segmentation of organoids of varying sizes and to quantify multiple morphological parameters, including projected area, perimeter, axis length, eccentricity, circularity, roundness, and compactness. As these parameters correlate with organoid viability, they provide a quantitative basis for assessing the inhibitory effects of drug treatment on organoid growth. In this context, automated comparison of pre- and post-treatment parameters enables objective calculation of drug inhibition rates, thereby improving the reliability of efficacy evaluation in preclinical drug development [43].

Furthermore, the integration of AI with multi-omics technologies has generated two major advances in this field. First, it has transformed organoids from a “single phenotypic screening tool” into a “multidimensional mechanism analysis platform”. For instance, organoid-based systems can recapitulate in vivo epigenetic regulatory processes, thereby providing a robust platform for the investigation of drug–epigenetic interactions [52]. Second, the establishment of databases such as OrgXenomics [53] has addressed the issue of “organoid data silos” and created essential resources for drug repurposing across diverse cancer types. Notably, in immuno-oncology, the integration of AI has enhanced prediction accuracy and optimized therapeutic strategies, enabling rigorous evaluation of the efficacy of immunotherapies such as immune checkpoint inhibitors, CAR-T cell therapies, and tumor vaccines [121].

### 7.2. Automation and High-Throughput Technologies Accelerate Drug Development Efficiency

Ma and colleagues employed microfluidic technology to generate uniform microdroplets from cell suspensions, thereby overcoming the screening biases introduced by size heterogeneity in manually cultured organoids. Importantly, this approach enables precise regulation of drug exposure and culture conditions with high spatiotemporal resolution. By integrating serial dilutions within microfluidic chips, automated gradient drug administration can be achieved, providing robust technical support for systematic drug screening [56]. In parallel, Zhang et al. developed a lung cancer organoid assembloid model using droplet microfluidic technology combined with a microinjection strategy. This platform demonstrated the capacity to accurately predict clinical outcomes in patients undergoing neoadjuvant immunotherapy in combination with chemotherapy and targeted therapy, highlighting its translational value in guiding personalized treatment strategies [55]. Specifically, the LCAs generated by the research team accurately recapitulated therapeutic responses to immune checkpoint inhibitor PD-1 combined with platinum-based doublet chemotherapy. The findings indicated that this combination regimen exhibited superior antitumor efficacy compared with either platinum-based doublet chemotherapy or PD-1 inhibitor monotherapy. Moreover, the LCAs showed higher sensitivity to the “PD-1 inhibitor + TC regimen (Taxol + Carboplatin)” compared with the TC regimen alone. These results were consistent with clinical observations, in which corresponding patients exhibited significant tumor shrinkage following two cycles of treatment with the “PD-1 inhibitor + TC regimen”.

The core value of automated technologies, such as the ReBiA system, lies in advancing organoids from specialized laboratory tools to industrialized platforms for drug screening. This system enhances reproducibility, standardization, production capacity, and cost-effectiveness, thereby addressing key limitations of conventional organoid culture. Organoid models generated by this platform are expected to serve as robust references for disease modeling, large-scale drug screening in pharmaceutical companies, and applications in personalized medicine [62]. Notably, the platform developed by Boussaad et al. incorporates “2D/3D compatibility”, enabling parallel drug evaluation in both “target cell models”, e.g., disease-relevant cells, and “normal cell/organoid models”. Its high-throughput screening (HTS) functionality supports simultaneous assessment of therapeutic efficacy and toxicity, thereby eliminating compounds with adverse effects on normal tissues at an early stage. This dual-assessment approach provides a reliable screening tool for translational research and precision medicine [70]. In this context, high-throughput bioprinting technologies further expand the capacity to generate reproducible organoid models, with excellent dimensional uniformity, achieving a coefficient of variation (CV) for size below 10%. Proof-of-concept studies involving five renal cell carcinoma (RCC)-targeted drugs demonstrated that the heterogeneity of organoids can be directly correlated with patients’ therapeutic responses [122]. For pharmaceutical companies, high-throughput automation not only accelerates large-scale drug development by enabling synchronous testing of extensive compound libraries and shortening preclinical timelines but also facilitates rapid identification of new indications for approved drugs, thereby advancing clinical translation and maximizing the therapeutic value of existing compounds.

### 7.3. Immune Co-Culture Systems Facilitate the Development of Immunotherapeutic Drugs

The co-culture of tumor organoids with immune cells has represented a pivotal advancement in faithfully reconstructing the dynamic interactions and regulatory networks within the TME [80]. This system has emerged as a critical technological platform supporting immunotherapeutic drug development. Its applications extend beyond high-throughput screening to encompass mechanistic analyses of therapeutic responses, rational design of combination strategies, and personalized efficacy prediction. Collectively, these capabilities establish an essential translational bridge from preclinical research to clinical application, thereby accelerating the development and optimization of next-generation immunotherapies. In liver cancer research, organoid-on-a-chip models constructed from PDOs can recapitulate the immune microenvironmental characteristics of native tumors, thereby providing a standardized and high-throughput platform for screening anti-liver cancer immunotherapeutic drugs. By enabling functional evaluation of candidate immunotherapeutics, this system has demonstrated screening outcomes that show strong concordance with the clinical treatment responses of corresponding patients. In particular, one study focused on PD-1/PD-L1 immunotherapy and Atezolizumab (an anti-PD-L1 antibody), showing that the co-culture model offered greater suitability than conventional PDO models for in vitro testing of immunotherapeutic drugs. Notably, this approach enhances the accuracy and reliability of preclinical-to-clinical translation, supporting the development of more effective immunotherapeutic strategies for liver cancer [123].

The utility of this system extends beyond drug screening, serving as a critical tool for elucidating immunotherapeutic mechanisms and refining combination strategies. For instance, in a triple co-culture model comprising gastric cancer organoids, CAFs, and NK cells, studies demonstrated that CAFs induce ferroptosis in NK cells via iron transport pathways, thereby suppressing their anti-tumor cytotoxic activity [80]. This finding not only reveals a novel stromal cell-mediated immune escape mechanism in gastric cancer but also identifies a therapeutic target for clinical development of synergistic regimens combining immunotherapy with iron chelators, deferoxamine (DFO) and deferiprone (DFP), offering a promising strategy to overcome immunotherapy resistance in specific patient subgroups. In cholangiocarcinoma, the co-culture of PDOs with autologous immune cells further underscores the translational potential of this approach for personalized immunotherapy [75]. As a proof-of-principle model, this system provides two essential functions: first, enabling in vitro assessment of the anti-tumor activity of novel ICIs and generating quantitative data for preclinical drug evaluation; second, leveraging inter-organoid variability in ICI responses to identify the most suitable inhibitor for individual patients, thereby predicting potential clinical benefit and supplying verifiable in vitro evidence to guide the design of personalized treatment regimens for cholangiocarcinoma.

### 7.4. Vascularized Organoid Models Optimize Drug Delivery and Toxicity Assessment

Traditional organoid systems are limited in their ability to replicate in vivo drug distribution and to accurately assess toxicity, largely due to the absence of functional vascular networks. As an important direction within the “Organoid plus” technology framework, vascularized organoid models enhance the physiological relevance of preclinical testing by recapitulating organ-specific vascular structures and microenvironments. In this context, such models provide critical support for optimizing drug delivery strategies, improving the prediction of bioavailability and pharmacokinetics, and refining toxicity assessment. Notably, the integration of vascularized organoids with advanced drug formulation approaches enables more accurate evaluation of in vivo performance and therapeutic windows, thereby strengthening their translational value for pharmaceutical development.

Banaeiyan et al. developed a vascularized liver cancer organoid-on-a-chip capable of replicating the microenvironmental characteristics of hepatic lobules. This platform employs a tissue-mimetic hexagonal design and incorporates microchannels to reproduce the convection–diffusion mechanisms of blood circulation. Such liver-lobule-on-a-chip microphysiological systems hold significant promise for drug development and hepatotoxicity studies by overcoming the limitations of non-vascularized organoids in simulating in vivo drug delivery dynamics. Delivery performance can be quantitatively assessed through parameters such as flow velocity, shear stress, glucose diffusion efficiency, and the Péclet Number (Pe) [124]. In this context, vascularized BBB organoid models further extend the applicability of this technology by enabling more accurate assessment of in vivo drug penetration rates. For example, the study examined BKM120, a molecule with known BBB permeability [125,126], and dabrafenib, which has limited BBB penetration [127]. As expected, high concentrations of BKM120 were detected in BBB organoids, whereas dabrafenib was undetectable. Additionally, concentrations of angiopep-2 (a peptide ligand) and its corresponding scrambled control peptide were measured, yielding results consistent with their expected BBB permeability profiles. Notably, these models are readily scalable to high-throughput formats, making them particularly suitable for investigating brain-penetrant molecules and supporting broad-spectrum therapeutic research in central nervous system disorders [128].

Vascularized organoids not only enhance the evaluation of drug efficacy [119] but also advance drug toxicity research. By closely recapitulating the 3D architecture and microenvironment of native tissues, they serve as robust in vitro models for pharmacology, toxicology, and drug discovery [108]. In this context, recent advances in organoid platforms incorporating microtissue vascularization and biological barrier features enable more precise investigation of drug transport and barrier permeability. Notably, these models also provide a valuable basis for the development of innovative drug delivery strategies, including nanocarrier-based systems designed to cross critical in vivo barriers [129].

“Organoid plus” establishes a technological framework that more closely replicates physiological conditions and demonstrates greater translational relevance for drug research and development through the multidimensional integration of computational approaches, automation technologies, immune co-culture systems, and vascularized models. This paradigm addresses the critical limitations of traditional drug development, namely, low screening efficiency and limited predictive accuracy, by providing robust platforms for mechanistic investigation and efficacy evaluation. In this context, “Organoid plus” not only accelerates the progression of precision medicine from concept to clinical application but also offers essential technical support for the development of patient-centered, personalized therapies. Ultimately, this approach is expected to transform the landscape of therapeutic drug development for major diseases, including cancer.

## 8. After the Hype: The Challenges and Future of Organoids

Organoid technology has emerged as a transformative platform in precision oncology, providing exceptional capabilities to replicate tumor biology and predict therapeutic responses. By preserving the histopathological and molecular complexity of primary tumors, organoids effectively bridge the gap between conventional preclinical models and clinical applications [6,130]. Nevertheless, the field encounters considerable challenges in areas such as standardization, microenvironment mimicry, and clinical translation. This SWOT analysis examines the strengths, weaknesses, opportunities, and threats influencing the development and application of organoid technology in oncology research and therapeutic strategies.

### 8.1. Strengths

Organoid technology offers significant advantages due to its biological fidelity and technological adaptability. Organoids facilitate clinically relevant drug screening and personalized therapeutic predictions [6,130]. Notably, recent progress in AI [56,62], immunotherapy research [79,131], and vascularization strategies [94,108] has further expanded the utility of these systems. Beyond these foundational capabilities, organoids uniquely recapitulate dynamic microenvironmental crosstalk, a feature largely absent from conventional models, thereby enabling more rigorous mechanistic investigations spanning preclinical drug discovery to translational research. In this context, the integration of multi-omics analytics enhances the resolution of therapeutic response profiling, providing deeper insights into drug–disease interactions. Collectively, these advancements position organoids as versatile platforms for refining drug development paradigms, overcoming persistent barriers in modeling in vivo physiological complexity, and accelerating the translation of preclinical findings into clinically actionable applications.

### 8.2. Weaknesses

Despite its considerable potential, organoid technology faces limitations that restrict its clinical translatability. Technical barriers [48], particularly in resource-constrained laboratories, reduce accessibility and scalability [62]. Notably, its application to neurovascular function studies and systemic drug metabolism research remains limited, restricting its utility in modeling complex physiological processes relevant to drug development. In alveolar lung injury cultures [79], the short-lived functionality of immune components further constrains the ability to conduct long-term evaluations of immunotherapy efficacy. In this context, these limitations highlight the urgent need for methodological innovations to establish more robust, scalable, and physiologically representative organoid models capable of supporting translational research and therapeutic development.

### 8.3. Opportunities

The potential of organoid technology is expansive, driven by interdisciplinary convergence and its translational applicability. To fully realize this potential, several key frontiers must be addressed, as summarized in Table 1. These include enhancing the physiological fidelity of models through improved recapitulation of native microenvironments, establishing robust standardization and scalability for high-throughput applications, and integrating organoids with advanced computational and engineering platforms. Such integration is pivotal for deepening mechanistic insights into disease processes and therapeutic responses. In parallel, accelerating clinical translation requires the development of structured biobanks linked to patient data, thereby bridging the gap between preclinical modeling and personalized therapeutic strategies. Ultimately, this multifaceted evolution demonstrates that the conventional conception of organoids is increasingly insufficient. Instead, their convergence with emerging technologies, the essence of the Organoid plus paradigm, has become indispensable for generating predictive, high-fidelity models in biomedical research and drug discovery. Realizing these opportunities will necessitate sustained cross-disciplinary collaboration to harness the full potential of organoids in pharmaceutical innovation. Notably, this convergence, central to the Organoid plus framework, establishes organoids as a cornerstone of next-generation preclinical modeling and translational drug development.

### 8.4. Threats

The advancement of organoid technology, while highly promising, remains contingent on overcoming several critical challenges. These obstacles, spanning technical, ethical, predictive, and competitive dimensions (as detailed in Table 1), pose substantial barriers to reproducibility, scalability, and eventual clinical translation. In this context, addressing such limitations will necessitate targeted investment in interdisciplinary collaboration, alignment with evolving regulatory frameworks, and the establishment of robust infrastructure to support the systematic validation and sustained evolution of organoid-based models.

### 8.5. The “Plus and Minus”: Internal Refinement and External Enhancement

**Figure 3:**
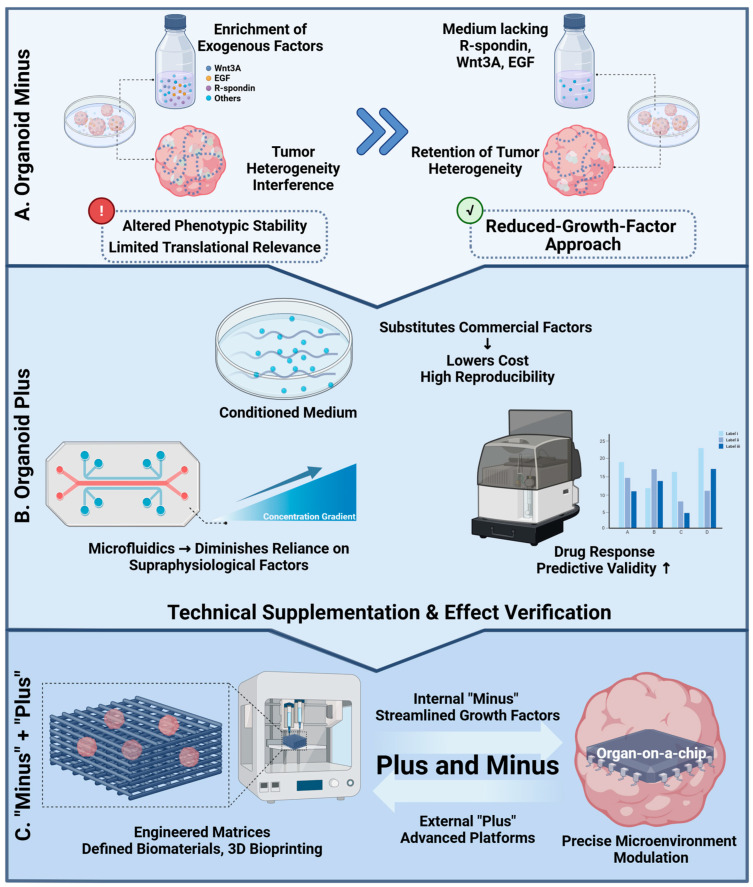
The “Plus and Minus” Strategies in Organoid Culture. (**A**) Organoid Minus. Conventional organoid culture relies on the supplementation of multiple exogenous growth factors, which may reduce phenotypic stability and compromise the preservation of tumor heterogeneity. The “minus” strategy streamlines growth factor requirements to better maintain tumor heterogeneity and enhance experimental reproducibility. (**B**) This strategy integrates conditioned medium with OoC or microfluidic platforms to more closely recapitulate the in vivo TME, thereby increasing the predictive relevance of organoid models for pharmacological response assessment. (**C**) Minus and plus. By simultaneously optimizing intrinsic growth factor composition and incorporating advanced external platforms, this combined approach enables precise and reproducible organoid culture systems with high translational potential for drug discovery and development.

As discussed earlier, solely emphasizing the supplementation or withdrawal of growth factors is insufficient to advance organoid culture systems. Future progress in organoid technology is expected to rely on a synergistic “Organoid Plus and Minus” strategy, as schematically illustrated in Figure 3. This framework encompasses the “minus” component within the culture system, streamlining the use of exogenous growth factors to preserve critical tissue-specific characteristics, and the external “plus” component, which integrates advanced technological platforms to construct a more precisely controllable biomimetic microenvironment. In this context, striking a balance between “internal optimization” and “external enhancement” is essential to transform organoids from basic in vitro culture models into high-fidelity physiological simulators, thereby strengthening their translational relevance for preclinical drug development.

## 9. Discussion and Perspectives

Organoids represent a transformative in vitro model system, gaining substantial research investment due to their potential applications in tumor biology, drug development, and precision medicine. These models are especially valuable in investigating tumorigenesis, screening anticancer drug candidates for both efficacy and toxicity, and guiding personalized therapeutic strategies. Their ability to model patient-specific tumors with high biological fidelity underscores their promise in advancing personalized cancer therapy. However, the functional and structural fidelity of tumor organoids has sometimes been overestimated in preclinical studies, with insufficient acknowledgment of their limitations in replicating the complexity of native TMEs. Despite this, organoids offer a unique advantage through their capacity for integration with complementary platforms, such as microfluidics, single-cell omics, and AI, which could enhance their applicability in precision oncology.

Future advancements should prioritize the integration of organoids with multidisciplinary technologies to achieve context-specific improvements. Such hybrid strategies can selectively optimize critical parameters, including accuracy, throughput, and model complexity, thereby addressing diverse research needs in drug discovery and translational oncology. Notably, while organoids provide unparalleled biological fidelity, their practical application is constrained by challenges in standardization and functional complexity, which require urgent resolution. In this context, leveraging opportunities in interdisciplinary integration, clinical validation, and automation will be essential to overcoming these barriers. Strategic emphasis on AI–driven analytics, regulatory harmonization, and technological innovation will position organoids as indispensable tools for drug development and precision oncology.

The convergence of emerging technologies has fostered the development of advanced organoid-based models. Within this context, the “Organoid minus” strategy emphasizes internal optimization through the simplification and refinement of culture systems. The adoption of minimalist, chemically defined media and engineered scaffolds seeks to uncover the intrinsic self-organization capacity of cells, thereby preserving authentic pathological features of tissues and improving the predictive validity of drug response data [34,35]. Complementing this approach, the “Organoid plus” strategy represents the next frontier in cancer research and therapeutic development. Unlike conventional organoids that primarily rely on biological fidelity, Organoid plus is defined by the systematic integration of five interdisciplinary pillars: AI, automated biofabrication, multi-omics analysis, vascularization strategies, and immune co-culture platforms, each addressing key limitations of traditional systems. Building upon the foundation of organoid technology, this paradigm enhances physiological relevance, standardization, scalability, and clinical applicability, thereby establishing a more precise research and application platform that better replicates the in vivo physiological environment.

Notably, the future advancement of organoid technology does not depend on either the “plus” or “minus” strategy alone, but rather on their deliberate integration. This synergistic “Organoid plus and minus” framework combines external technological augmentation with internal system optimization. By leveraging advanced engineering and computational platforms to construct tightly regulated microenvironments, it enables the application of low-factor culture conditions that more closely approximate physiological contexts. In this way, the integrated framework provides a balanced approach to maintaining fidelity, scalability, and reproducibility, ultimately supporting the translation of organoid models into clinically actionable tools for precision oncology and pharmaceutical development [34,54,61].

Despite significant advances, persistent limitations continue to impede the clinical translation of organoid technology. Challenges such as batch-to-batch variability [132] and the incomplete replication of stromal-immune microenvironments [54] remain critical barriers. Additionally, the resource-intensive nature of AI integration and multi-omics analytics [48,62] restricts accessibility, particularly in resource-constrained environments. These issues highlight the urgent need for standardized protocols and cost-effective solutions to broaden the applicability of organoid-based models. The future of organoid technology depends on deep interdisciplinary integration. Microfluidics-enabled organoid-on-a-chip platforms [61,118] and robotic automation systems [62] offer promising avenues for dynamic modulation of the TME and real-time monitoring, both of which could enhance predictive accuracy in preclinical models. Furthermore, co-clinical trials and organoid biobanking initiatives could facilitate biomarker validation and expedite regulatory approvals for stroma-targeted therapies. The development of vascularized multi-organ chips represents a novel approach to modeling systemic drug responses and metastasis, providing valuable insights into complex disease states and advancing the understanding of drug efficacy in multi-organ contexts.

Despite significant progress, ethical concerns regarding stem cell use [104] and intellectual property disputes [108] continue to present potential barriers that must be carefully navigated to foster innovation and the widespread adoption of organoid technologies.

Organoid technology represents a paradigm shift in precision oncology, bridging the gap between conventional models and clinical realities. Its ability to preserve tumor heterogeneity and patient-specific molecular signatures positions it as a cornerstone for personalized drug discovery [6,130]. However, challenges related to standardization, microenvironment replication, and scalability persist [54,132]. In this context, strategies centered on internal optimization of culture systems and external technological integration provide a viable path to address these limitations. Strategic investments in protocol harmonization, interdisciplinary collaboration, and infrastructure development are essential to realizing the full potential of organoid platforms. By synergizing in vitro, in vivo, and in silico approaches, organoids will play a critical role in accelerating the development of tailored therapies for stroma-rich malignancies, ultimately improving clinical outcomes for cancer patients worldwide.

## 10. Conclusions

This review systematically examines the applications and challenges of organoid technology in addressing tumor heterogeneity and advancing innovative strategies for drug efficacy assessment. We propose the “Organoid plus and minus” paradigm, which emphasizes enhancing the physiological relevance, standardization, and translational potential of organoid models through the integration of interdisciplinary technologies, including AI, automated biomanufacturing, multi-omics profiling, immune co-culture systems, and vascularization strategies. Although organoids provide distinct advantages in maintaining the architectural and molecular features of primary tumors, challenges remain regarding standardization, comprehensive recapitulation of the TME, and scalability for widespread application. Future progress in organoid technology will depend on a synergistic strategy of “internal refinement”, such as optimizing medium composition and minimizing dependence on exogenous factors, and “external enhancement”, exemplified by the incorporation of microfluidic systems and bioengineered scaffolds. In this context, fostering multidisciplinary collaboration, establishing standardized biobanks, and pursuing co-clinical validation will be critical for maximizing the translational utility of organoid platforms. Notably, organoids hold substantial promise as predictive tools for personalized drug screening and efficacy evaluation, thereby accelerating the development of precision oncology therapeutics and supporting innovation in cancer drug discovery.

## Figures and Tables

**Table 1:** Organoids: SWOT analysis of key aspects and characteristics.

Category	Aspect	Key Characteristics	References
Strengths	Biological fidelity	Retains 3D architecture, cellular heterogeneity, and native tumor molecular profiles Facilitating more clinically relevant drug screening	[6,43,130]
	Technological integration	AI drug sensitivity modeling Automated platforms for reproducibility or scalability	[43,44,56,62]
	Immunotherapy applications	Preserves TILs, supports CAR-T/NK interaction studies Optimizing immune cell therapies	[79,131]
	Vascularization strategies	Stem cell co-differentiation and bioengineered scaffold Enabling perfusable vascular networks and enhancing TME mimicry	[94,108]
Weaknesses	Technical limitations	Batch variability and incomplete stromal/immune microenvironment replication	[132]
	Resource constraints	High costs in AI, multi-omics, and robotic automation	[48,62]
	Functional deficiencies	Lack of critical vasculature, neural networks, and long-term immune viability Limiting organoid application scope	[54,79,106]
Opportunities	Technological convergence	Microfluidics, robotics, AI integration Large-scale drug screening/therapeutic testing	[61,118]
	Clinical translation	Biobanking and co-clinical trials Opportunities for biomarker validation and personalized drug screening	[41,133]
	Advanced modeling platforms	Vascularized multi-organ chips, accurate metastasis/BBB modeling	[110,112]
	Regulatory advancement	Protocol standardization: accelerating FDA/EMA approval Ensuring faster organoid clinical adoption	
Threats	Technical barriers	Robotic precision and multi-omics integration challenges Hindering reproducibility/scalability	[48,70]
	Ethical/legal concerns	Stem cell ethics, scalability, and IP disputes impeding adoption/innovation	[104,108]
	Predictive validity challenges	Organoid-clinical outcome discrepancies undermining clinician confidence	[83,134]
	Competitive technologies	The rise in OoC and PDX, potential competition for organoids	[109,135]

## Data Availability

No new data were created or analyzed in this study. Data sharing is not applicable to this article.

## Abbreviations

The following abbreviations are used in this manuscript:

2D	Two-dimensional
3D	Three-dimensional
AI	Artificial Intelligence
ALI	Air–Liquid Interface
ASC	Adult Stem Cell
BBB	Blood–Brain Barrier
BME	Basement Membrane Extract
CAR-T	Chimeric Antigen Receptor T-cell
CAF	Cancer-Associated Fibroblast
CRCO	Colorectal Cancer Organoids
DC	Dendritic Cell
DL	Deep Learning
EC	Endothelial Cell
ECM	Extracellular Matrix
FDA	Food and Drug Administration
HCC	Hepatocellular Carcinoma
HUVEC	Human Umbilical Vein Endothelial Cell
hPSC	Human Pluripotent Stem Cell
iPSC	Induced Pluripotent Stem Cell
LCA	Lung Cancer Assembloid
ML	Machine Learning
NK	Natural Killer
OoC	Organ-on-a-Chip
PBMC	Peripheral Blood Mononuclear Cell
PDO	Patient-Derived Organoid
PDX	Patient-Derived Xenograft
ReBiA	Robotic-Enabled Biological Automation
TAM	Tumor-Associated Macrophage
TIL	Tumor-Infiltrating Lymphocyte
TME	Tumor Microenvironment
VEGF	Vascular Endothelial Growth Factor
